# Efficacy and safety of Ixazomib-based maintenance therapy after autologous hematopoietic stem cell transplantation in multiple myeloma patients: a retrospective analysis

**DOI:** 10.3389/fmed.2026.1680704

**Published:** 2026-01-29

**Authors:** Mengyuan Chen, Gang Chen, Jianli Xu, Kaile Zhang, Ruixue Yang, Chunxia Han, Jia Hou, Ming Jiang, Hailong Yuan

**Affiliations:** Department of Hematology, The First Affiliated Hospital of Xinjiang Medical University, Urumqi, China

**Keywords:** autologous hematopoietic stem cell transplantation, Ixazomib, maintenance therapy, MRD, multiple myeloma

## Abstract

**Introduction:**

The therapeutic standard for post-autologous stem cell transplantation (ASCT) maintenance in multiple myeloma (MM) is evolving, with daratumumab-based regimens emerging as highly effective. However, access to such therapies remains limited in regions like China. This study evaluated the real-world efficacy and safety of the oral proteasome inhibitor ixazomib as a practical alternative for maintenance therapy in this setting.

**Methods:**

This single-center, retrospective analysis included 28 MM patients who received ixazomib-based maintenance (monotherapy or combination) for ≥5 months after ASCT (from a total cohort of 64 ASCT recipients) between August 2019 and August 2023. Treatment was assigned based on mSMART 3.0 risk stratification and adjusted per clinical circumstances. The primary endpoint was progression-free survival (PFS). Secondary endpoints included treatment response, minimal residual disease (MRD) status, and safety.

**Results:**

The median age of the patients was 57 years (range: 48–69). Before ASCT, 9 patients (32.1%) achieved complete response (CR), 7 (25.0%) achieved very good partial response (VGPR), and 12 (42.9%) achieved partial response (PR). After ASCT, responses deepened: 17 patients (60.7%) achieved CR, 6 (21.4%) VGPR, and 5 (17.9%) PR. Following Ixazomib maintenance, 15 patients (53.5%) were in CR, 8 (28.6%) in VGPR, and 5 (17.9%) in PR. Four patients experienced disease progression during follow-up. The incidence of grade ≥ 3 adverse events was 3.6%. The estimated 3-year PFS and overall survival rates were 70.8% (95% CI, 52.18%–89.42%) and 87.4% (95% CI, 73.88%–100.92%), respectively. Notably, patients who sustained MRD negativity for over 1 year had an estimated 3-year PFS of 100%, significantly higher than that of MRD-positive patients.

**Discussion:**

Within the constraints of the current Chinese clinical context, ixazomib-based maintenance therapy demonstrates favorable efficacy and a manageable safety profile for MM patients after ASCT, offering a viable and accessible alternative. Achieving sustained MRD negativity is critically important for long-term outcomes, reinforcing its value as a key treatment goal. These real-world findings support the role of ixazomib, particularly for patients ineligible for or intolerant to standard regimens.

## 1 Introduction

Multiple myeloma (MM) is a malignant clonal plasma cell disorder and the second most common hematologic malignancy worldwide (1). The incidence of MM has been increasing annually, and many countries, including China, are facing an increasing burden of disease. From 1990 to 2021, the age-standardized incidence rate of MM in China increased by 3.1%, the mortality rate rose by 2.2%, and the prevalence significantly increased by 5.8% (2). Despite the ongoing development of new drugs and treatment protocols (3–5), autologous hematopoietic stem cell transplantation (ASCT) remains the first-line consolidation treatment recommended by international guidelines, including those from the American Society of Clinical Oncology (6) and the European Society for Medical Oncology (7), for transplant-eligible MM patients. It is also considered the standard treatment for newly diagnosed MM patients meeting the eligibility criteria, significantly deepening responses and improving progression-free survival (PFS) (8–10). The pivotal role of ASCT in prolonging PFS has been reaffirmed by recent landmark randomized controlled trials (11) and is supported by extensive real-world data from global registries (12). Despite its established efficacy, disease relapse post-ASCT remains a major challenge, making effective maintenance therapy critical for prolonging remission and overall survival (OS).

The landscape of post-ASCT maintenance is rapidly evolving. Lenalidomide monotherapy has long been the established standard (13, 14). Recently, this paradigm has been challenged and advanced by the integration of anti-CD38 monoclonal antibodies. Landmark trials such as PERSEUS have demonstrated the superior efficacy of daratumumab combined with lenalidomide (Dara-R), setting a new benchmark for maintenance therapy in many regions (15). Concurrently, the role of proteasome inhibitor (PI)-based maintenance, particularly in high-risk cytogenetic abnormality (HRCA) patients, continues to be explored (16, 17). In this context, the oral PI Ixazomib offers a compelling alternative due to its convenient administration, favorable toxicity profile, and potential for combination strategies. However, its positioning requires careful consideration. Indirect comparisons of clinical trial data suggest that while Ixazomib maintenance provides a significant PFS benefit over placebo, its effect magnitude is generally considered more modest than that achieved with lenalidomide maintenance (18, 19). This has prompted a reevaluation of Ixazomib’s niche, shifting focus towards its utility in specific subgroups, such as HRCA patients, or as an alternative for those intolerant to standard regimens.

Importantly, the translation of global therapeutic advances into clinical practice is heavily influenced by regional factors, including drug approval status and reimbursement policies. In China, while novel agents are increasingly available, daratumumab is not yet approved or reimbursed for post-ASCT maintenance therapy, rendering this potent regimen inaccessible for the vast majority of patients due to prohibitive out-of-pocket costs. In contrast, Ixazomib is both approved and covered by national health insurance, making it a feasible and sustainable option for long-term maintenance. Furthermore, the injectable PI bortezomib is limited in the maintenance setting by cumulative toxicities, notably peripheral neuropathy (20). Ixazomib, as the first oral PI, addresses key limitations of traditional injectable PIs by offering improved convenience and patient adherence (21). Early studies, such as IFM 2013-06 and MMRC-066, suggested its safety and potential efficacy in the post-ASCT maintenance setting (18, 22). However, despite growing real-world evidence from other regions (23, 24), its long-term effectiveness, safety, and optimal role within the specific constraints of the Chinese healthcare system, particularly in relation to cytogenetic risk and minimal residual disease (MRD) dynamics, remain inadequately characterized.

Therefore, this single-center, retrospective study aims to address this gap by analyzing the outcomes of MM patients receiving Ixazomib-based maintenance therapy after ASCT at our institution. We seek to evaluate its real-world effectiveness and safety profile, and to explore the prognostic impact of sustained MRD negativity in this setting. By doing so, we hope to provide timely, hypothesis-generating evidence that can inform clinical decision-making and guide the design of future prospective studies in an evolving yet resource-conscious therapeutic landscape.

## 2 Methods

### 2.1 Study participants

A retrospective analysis was conducted on the clinical data of 64 consecutive patients with MM who underwent ASCT at the First Affiliated Hospital of Xinjiang Medical University, from August 2019 to August 2023. The inclusion criteria were as follows: (1) The diagnosis of MM was made according to the criteria established by the International Myeloma Working Group (IMWG) in 2016 (25); (2) Patients aged 18 to 70 years; (3) Patients who received ASCT and underwent post-transplant maintenance therapy. The exclusion criterion was a post-transplant maintenance therapy duration of less than 5 months. This study was performed in line with the principles of the Declaration of Helsinki and approved by the Ethics Committee of Xinjiang Medical University (approval no. K202501-37). Informed consent was obtained from all participants.

### 2.2 Induction therapy before ASCT

Among the 64 patients, 41 underwent induction treatment with the VRD regimen (bortezomib, lenalidomide, and dexamethasone), while 21 received the VTD regimen (bortezomib, thalidomide, and dexamethasone), and 2 were treated with the VCD regimen (bortezomib, cyclophosphamide, and dexamethasone). The 7 patients aged 65 and older were administered a three-drug combination induction regimen, including 3 patients on the VTD regimen and 4 on the VRD regimen. The median number of induction therapy cycles before ASCT for the 64 patients was 5.7 (range: 4–15).

### 2.3 Mobilization, collection, and preservation of autologous hematopoietic stem cells

Among 64 patients, 52 underwent a single mobilization of peripheral blood stem cells. Of these, 25 received a regimen of granulocyte colony-stimulating factor (G-CSF) combined with plerixafor, while 23 were treated with cyclophosphamide (CTX) and G-CSF. Additionally, three patients received a combination of CTX, G-CSF, and plerixafor, and one patient was administered a DECP (Dexamethasone, Etoposide, Carboplatin, and Cisplatin) regimen combined with G-CSF.

Twelve patients underwent two mobilizations, with all initially receiving a CTX plus G-CSF regimen. Among them, nine patients had a second mobilization with DECP and G-CSF, whereas three patients received a regimen of DECP, G-CSF, and plerixafor. The collected peripheral blood stem cells were subsequently mixed with the cell preservation solution and aliquoted for storage in a −80 °C freezer. For a single ASCT, it is recommended that the collected CD34^+^ cell count should preferably exceed 2 × 10^6^/kg.

### 2.4 Pre-conditioning regimen

Fifty-seven patients received a standard pre-conditioning regimen consisting of an intravenous dosage of melphalan at 200 mg/m^2^, while seven patients aged 65 years and older were administered an intravenous dosage ranging from 140 to 160 mg/m^2^. During the pretreatment period, patients were given prophylactic treatments for oral mucositis, diarrhea, and hepatic sinusoidal obstruction syndrome, along with appropriate treatment for those with comorbid conditions.

### 2.5 Post-ASCT maintenance therapy

Based on the mSMART 3.0 guidelines, we categorized 64 patients into cytogenetic risk groups once their blood parameters had stabilized (at 2 to 3 months post-ASCT) (26). The initial assignment to maintenance therapy was as follows: patients classified as standard-risk (SR) were primarily prescribed maintenance therapy with single-agent lenalidomide. Conversely, patients classified as having high-risk cytogenetic abnormalities (HRCA) were primarily prescribed a combination of lenalidomide and Ixazomib. However, subsequent adjustments to this regimen were permitted based on individual patient factors, including laboratory results (e.g., drug intolerance, hematologic toxicity), therapeutic responses (e.g., persistent positive MRD), and the clinician’s assessment of the disease course (e.g., emergence of clinical high-risk features). Ixazomib was administered orally at a dose of 4 mg on days 1, 8, and 15, while lenalidomide was given at 10 mg orally from days 1 to 21, with treatment being discontinued in cases of grade 3–4 hematological toxicity. Each treatment cycle lasted 28 days.

### 2.6 MRD detection

MRD assessment was performed using the first-generation flow cytometry following the EuroFlow standard operating procedures. The standardized 8-color antibody panel was designed to optimally identify and characterize aberrant plasma cells and included the following antibodies: CD19, CD27, CD38, CD45, CD56, CD81, CD138, and CytoPlasma (for cytoplasmic kappa and lambda light chains). The clonal plasma cells in the bone marrow without phenotypic abnormalities were detected, with a minimum detection sensitivity of one clonal plasma cell per 10^5^ nucleated cells. For patients who tested negative for MRD before ASCT, MRD detection was performed every 3 months (90 days); for those who tested positive, MRD detection was conducted every 2 months (60 days), and if two consecutive tests yielded negative results, the monitoring frequency was adjusted to every 3 months. Regular monitoring continued for a total of 2 years, after which monitoring was conducted every 6 months based on the patient’s condition.

### 2.7 Follow-up

Patients were followed up through outpatient visits, inpatient re-evaluations, social media, and telephone communications. The follow-up deadline was August 31, 2024, with no patients lost to follow-up. During follow-up, regular monitoring of MRD, serum-free light chains, and immunofixation electrophoresis was conducted. The adverse reactions were recorded. PFS was defined as the time from treatment initiation to disease progression, recurrence, or death. OS was defined as the time from definitive diagnosis to death or the end of follow-up.

### 2.8 Outcome measurements

The treatment efficacy was evaluated using the IMWG criteria (1), which categorized outcomes into stringent complete response, complete response (CR), very good partial response (VGPR), partial response (PR), stable disease, and progression. The safety of Ixazomib was assessed according to the Common Terminology Criteria for Adverse Events (CTCAE Version 5.0) (27). PFS was the primary outcome of the study, while the treatment efficacy and MRD status after ASCT were considered secondary outcomes.

### 2.9 Statistical analysis

Statistical analysis was conducted using SPSS version 26.0. Continuous variables are described using medians (range: minimum value-maximum value) and compared using the Mann–Whitney *U* test. Categorical variables are presented as frequencies (percentages). Intergroup comparisons were performed using the Chi-square test or the Fisher’s exact probability method, as appropriate. Survival analysis was conducted using the Kaplan–Meier method, and the differences in survival were compared with the Log-rank test. A *p*-value of <0.05 indicates statistical significance.

## 3 Results

### 3.1 Patient cohort and maintenance therapy

**Figure 1:**
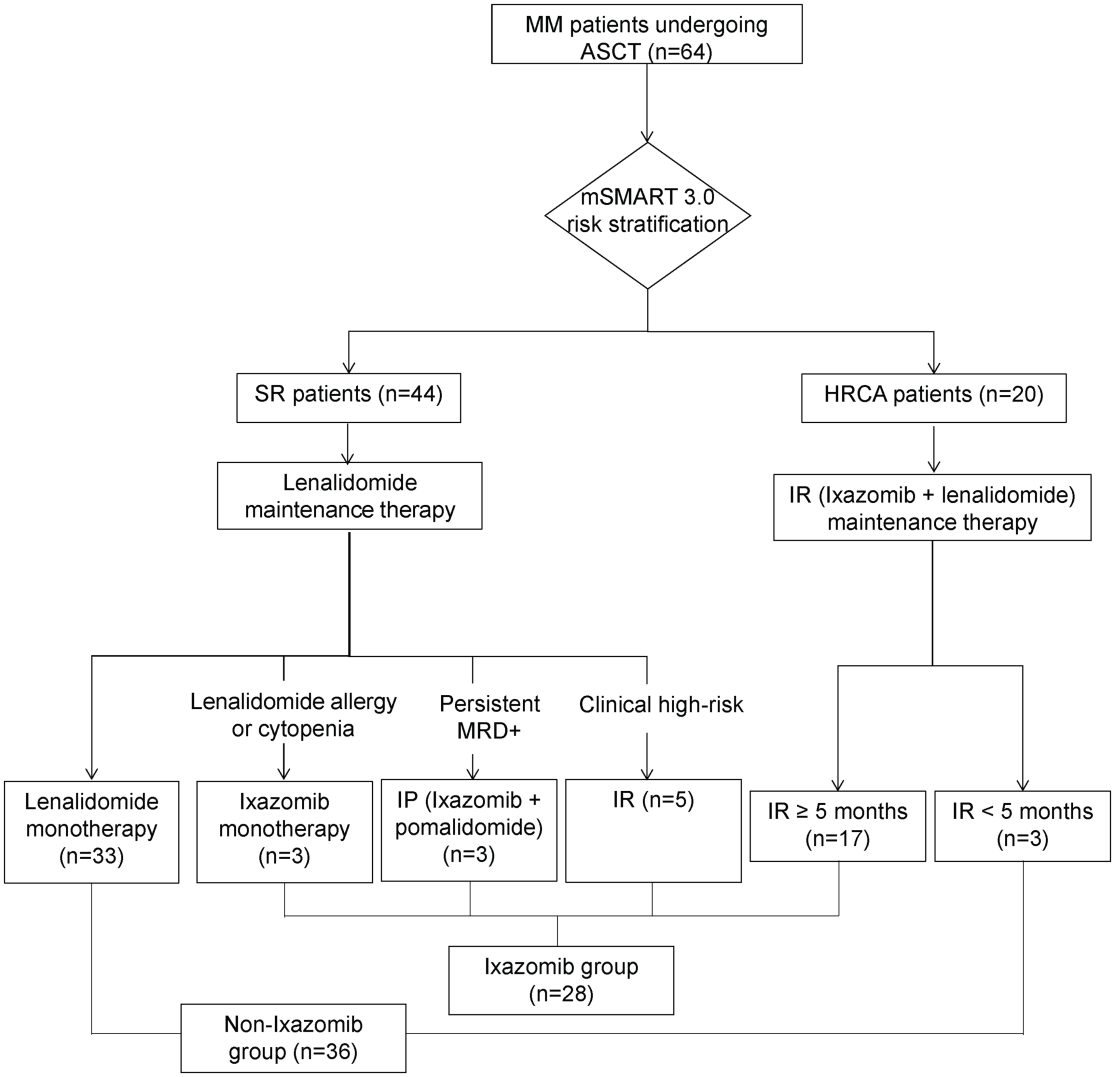
Flow diagram of the patient selection and maintenance therapy assignment. A total of 64 MM patients who underwent ASCT and received maintenance therapy were included. Based on mSMART 3.0 risk stratification, SR patients were primarily assigned to lenalidomide monotherapy, while HRCA patients were assigned to an Ixazomib-based regimen (Ixazomib + lenalidomide). Specific clinical reasons (allergy, cytopenia, persistent MRD positivity, clinical high-risk status) led to some SR patients receiving Ixazomib. Patients who received Ixazomib-based maintenance for ≥5 months formed the Ixazomib group (*n* = 28), while others formed the non-Ixazomib group (*n* = 36). MRD, minimal residual disease; MM, multiple myeloma; ASCT, autologous stem cell transplantation; SR, standard-risk; HRCA, high-risk cytogenetic abnormality.

The formation of the patient cohort and the assignment to maintenance therapy are summarized in Figure 1. In total, 64 patients who successfully underwent ASCT and subsequently received maintenance therapy were selected. Based on the mSMART 3.0 criteria, 44 patients were categorized as SR, and 20 patients were classified as HRCA. Among the 44 patients with SR, 33 received lenalidomide monotherapy, while 11 received Ixazomib-based therapy due to specific clinical circumstances (lenalidomide allergy or cytopenia, *n* = 3; clinical high-risk status, *n* = 3; persistent positive MRD, *n* = 5). Among the 20 patients with HRCA, 17 received the IR (Ixazomib + lenalidomide) regimen, while 3 discontinued Ixazomib within 5 months (due to thrombocytopenia or early relapse) and were thus excluded from the Ixazomib group. Consequently, the final analytic sets consisted of 28 patients in the Ixazomib group and 36 in the non-Ixazomib group. Within the Ixazomib group, the specific regimens included Ixazomib monotherapy (*n* = 3), IP (Ixazomib + pomalidomide, *n* = 3), and IR (*n* = 22). Among the 22 patients on the IR regimen, 3 patients switched to monotherapy with Ixazomib after maintaining persistent MRD negativity for over 2 years.

### 3.2 Patient baseline characteristics

The baseline clinical characteristics of the 28 patients in the Ixazomib group and the 36 patients in the non-Ixazomib group are summarized in Table 1. Intergroup comparisons showed no statistically significant differences in median age (57 vs. 58 years), gender distribution, immunoglobulin type, or R-ISS (Revised International Staging System) staging (all *p* > 0.05). Notably, a higher proportion of patients in the Ixazomib group had HRCA (60.7% vs. 8.3%, *p* < 0.001) and specifically, 1q + abnormalities (60.7% vs. 11.1%, *p* < 0.001). Importantly, the depth of response achieved immediately after ASCT was comparable between the two groups (*p* = 0.945), with ≥VGPR rates of 82.1 and 83.3%, respectively.

**Table 1:** Baseline characteristics of the Ixazomib and non-Ixazomib groups.

Variables	Ixazomib group (*n* = 28)	Non-Ixazomib group (*n* = 36)	*p*-value
Median age, years (range)	57 (48–69)	58 (45–70)	0.876
Gender, *n* (%)			0.974
Male	17 (60.7%)	22 (61.1%)	
Female	11 (39.3%)	14 (38.9%)	
Immunoglobulin type, *n* (%)			0.801
IgG type	17 (60.7%)	20 (55.6%)	
IgA type	6 (21.4%)	9 (25.0%)	
Light chain type	3 (10.7%)	5 (13.9%)	
Non-secretory type	2 (7.2%)	2 (5.6%)	
R-ISS staging, *n* (%)			0.912
Stage I	4 (14.3%)	6 (16.7%)	
Stage II	19 (67.9%)	24 (66.7%)	
Stage III	5 (17.9%)	6 (16.7)	
Cytogenetic risk stratification, *n* (%)			<0.001
Standard-risk cytogenetic abnormalities	11 (39.3%)	33 (91.7%)	
High-risk cytogenetic abnormalities	17 (60.7%)	3 (8.3%)	
1q status, *n* (%)			<0.001
1q + (Gain/Amp)	17 (60.7%)	4 (11.1%)	
No 1q+	11 (39.3%)	32 (88.9%)	
Response post-ASCT, *n* (%)			0.945
Complete response	17 (60.7%)	22 (61.1%)	
Very good partial response	6 (21.4%)	8 (22.2%)	
Partial response	5 (17.9%)	6 (16.7%)	
≥Very good partial response	23 (82.1%)	30 (83.3%)	

R-ISS, revised international staging system; ASCT, autologous hematopoietic stem cell transplantation.

Statistical comparisons were performed using the Chi-square test, Fisher’s exact test, or the Mann–Whitney *U* test, as appropriate.

### 3.3 Analysis of treatment efficacy

All 28 patients were evaluated for efficacy according to the IMWG criteria. As shown in Table 2, 9 patients achieved CR (32.1%), 7 achieved VGPR (25.0%), and 12 achieved PR (42.9%) before ASCT. After ASCT, 17 patients achieved CR (60.7%), 6 achieved VGPR (21.4%), and 5 achieved PR (17.9%). Three patients demonstrated an improvement from PR to VGPR after ASCT, while 4 patients improved from VGPR to CR, and another 4 improved from PR to CR. After maintenance therapy based on Ixazomib, 15 patients achieved CR (53.5%), 8 achieved VGPR (28.6%), and 5 achieved PR (17.9%). Two patients improved from PR to VGPR, and 1 improved from PR to CR. However, 4 patients experienced disease progression: 2 progressed from CR to PR, 1 from CR to VGPR, and 1 from VGPR to PR, all of whom were in relapse. Maintenance therapy based on Ixazomib after ASCT was beneficial for maintaining an effective depth of response (≥VGPR) in patients with MM.

**Table 2:** Treatment efficacy of 28 patients receiving maintenance therapy based on Ixazomib.

Treatment efficacy	CR	VGPR	PR	≥VGPR
Before ASCT	9 (32.1)	7 (25.0)	12 (42.9)	16 (57.1)
After ASCT	17 (60.7)	6 (21.4)	5 (17.9)	23 (82.1)
After maintenance therapy based on Ixazomib	15 (53.5)	8 (28.6)	5 (17.9)	23 (82.1)

Data are presented as *n* (%). ASCT, autologous hematopoietic stem cell transplantation; CR, complete response; VGPR, very good partial response; PR, partial response.

Given the heterogeneity of the Ixazomib-based maintenance regimens, a descriptive subgroup analysis was performed. The treatment efficacy for patients receiving Ixazomib monotherapy (*n* = 3), IR (*n* = 22), and IP (*n* = 3) is summarized in Table 3. Although the small sample size in each subgroup limits statistical conclusions, the depth of response and the distribution of progression events suggest that the treatment effect was consistent across the different Ixazomib-based strategies in this cohort.

**Table 3:** Descriptive subgroup analysis of Ixazomib-based maintenance regimens.

Maintenance regimen	Patients (*n*)	Best response post-maintenance, *n* (%)				Relapse events, *n* (description)	Death events, *n* (cause)
		CR	VGPR	PR	≥VGPR		
Ixazomib + lenalidomide (IR)	22	12 (54.8%)	6 (27.3%)	4 (18.2%)	18 (81.8%)	5 • 2 extramedullary relapse (HRCA) • 3 biochemical progression (SR)	3 • 2 due to primary disease (HRCA) • 1 from hepatitis B liver failure (SR)
Ixazomib + pomalidomide (IP)	3	2 (66.7%)	1 (33.3%)	0 (0%)	3 (100%)	1 • 1 marrow relapse (SR)	0
Ixazomib monotherapy	3	1 (33.3%)	1 (33.3%)	1 (33.3%)	2 (66.7%)	0	0
Total	28	15 (53.6%)	8 (28.6%)	5 (17.9%)	23 (82.1%)	6	3

CR, complete response; VGPR, very good partial response; PR, partial response; SR, standard-risk; HRCA, high-risk cytogenetic abnormalities.

### 3.4 Analysis of safety

The median duration of Ixazomib treatment (≥5 months) in 28 patients was 23.25 months (ranging from 5 to 56 months). Fourteen patients were treated for more than 2 years, with 2 patients for over 4 years, and the longest duration was 56 months. During the treatment period, the safety profile was favorable. Common adverse reactions associated with Ixazomib-based maintenance therapy include hematologic toxicity, peripheral neuropathy, gastrointestinal symptoms, fatigue, tiredness, rash, insomnia, and drowsiness. In our cohort, the overall incidence of adverse events was 50%, with the highest incidence observed for gastrointestinal symptoms (28.6%) (Table 4). The rate of hematologic toxicity was 17.9%, including 4 cases of thrombocytopenia and 1 case of neutropenia. The incidence of grade ≥3 adverse reactions was 3.6%. Most patients tolerated these side effects well, and the symptoms improved following active symptomatic supportive care.

**Table 4:** Adverse reactions of maintenance therapy based on Ixazomib in 28 patients.

Adverse reactions		All grades	≥ Grade 3 adverse reactions
Gastrointestinal symptoms	Poor appetite	3 (10.7%)	—
	Diarrhea	5 (17.9%)	—
	Constipation	—	—
Hematologic toxicity	Anemia	—	—
	Thrombocytopenia	4 (14.3%)	1 (3.6%)
	Neutropenia	1 (3.6%)	—
Peripheral neuropathy		1 (3.6%)	—
Rash		—	—

Data are presented as *n* (%).

### 3.5 Survival and outcomes

As of the end of August 2024, the median follow-up time for 28 patients with MM was 28.5 months (ranging from 6 to 59 months). The median follow-up time for PFS was 25.8 months (ranging from 6 to 58.5 months), while the median OS follow-up time was 26.9 months (ranging from 7.5 to 58.5 months). Among the 28 patients, 6 experienced relapse (including 4 SR and 2 HRCA patients), and 3 died (2 HRCA patients from disease progression post-relapse, and 1 SR patient from hepatitis B-related liver failure unrelated to the primary disease). The detailed outcomes, including relapse and death events for each maintenance regimen subgroup, are described in Table 3. In the IR subgroup, 3 SR patients experienced biochemical relapse due to high clinical risk while on the lenalidomide regimen; their disease stabilized after switching to the IR regimen. Furthermore, 2 HRCA patients in this subgroup developed extramedullary relapse during IR maintenance and subsequently died from disease progression. This subgroup also included the aforementioned SR patient who died from unrelated hepatitis B-related liver failure. Within the IP subgroup, 1 SR patient with persistent MRD positivity suffered a bone marrow relapse but achieved clinical stability after transitioning to the IP regimen. No deaths occurred in this subgroup. In the Ixazomib monotherapy subgroup, 3 SR patients were switched to Ixazomib due to allergic reactions or cytopenia during treatment with the lenalidomide regimen. No further death events or relapses were reported after the transition.

**Figure 2:**
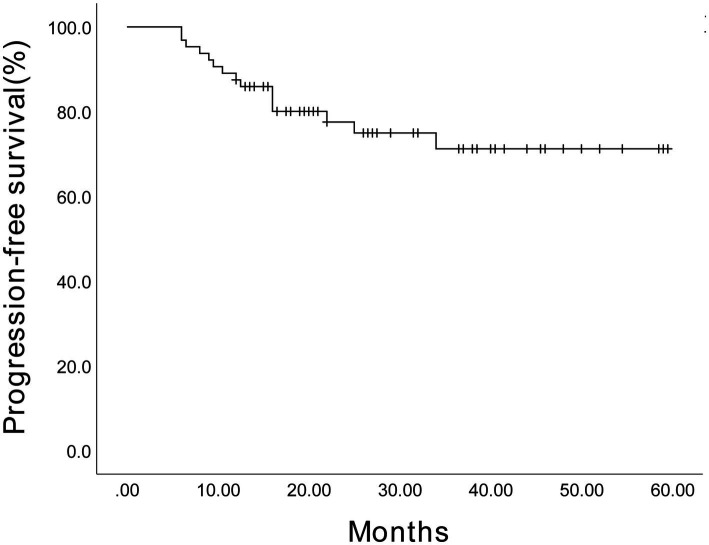
The Kaplan–Meier survival curve showing the estimated 3-year progression-free survival for 64 patients.

**Figure 3:**
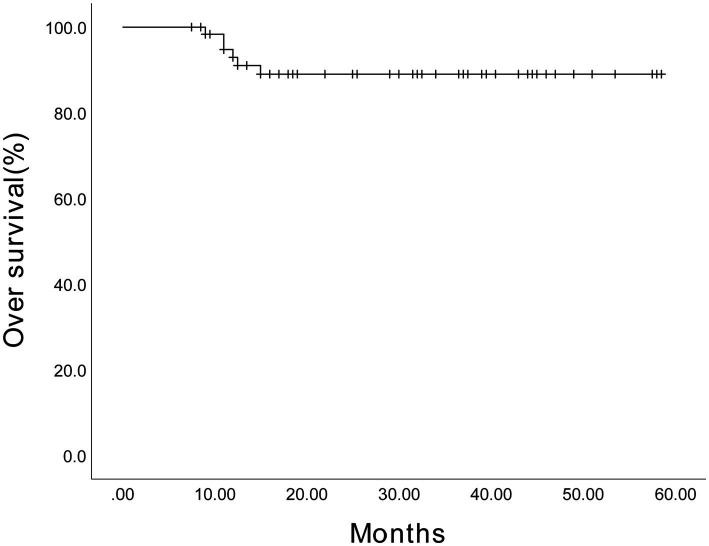
The Kaplan–Meier survival curve showing the estimated 3-year overall survival for 64 patients.

**Figure 4:**
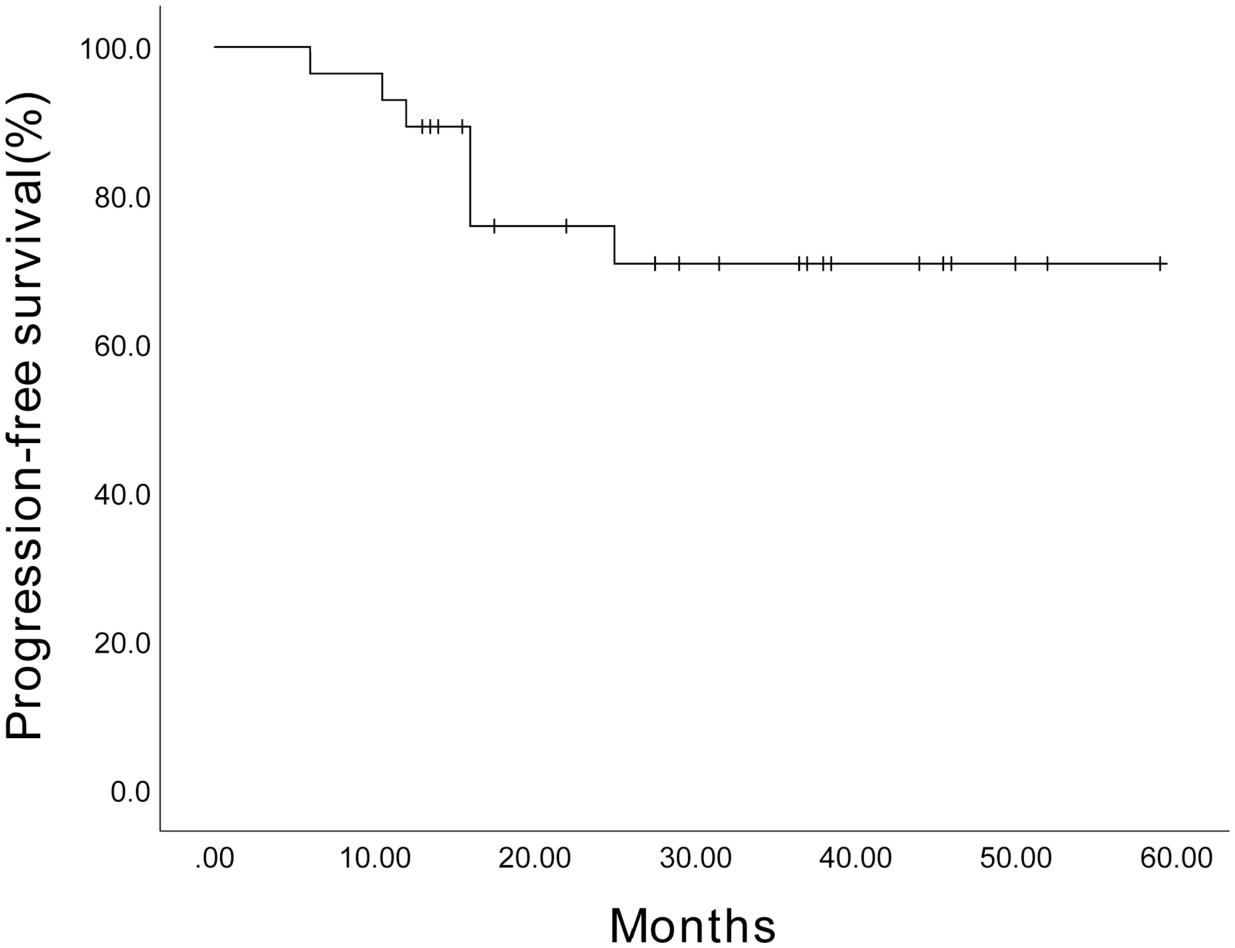
The Kaplan–Meier survival curve showing the estimated 3-year progression-free survival for 28 patients receiving maintenance therapy based on Ixazomib.

**Figure 5:**
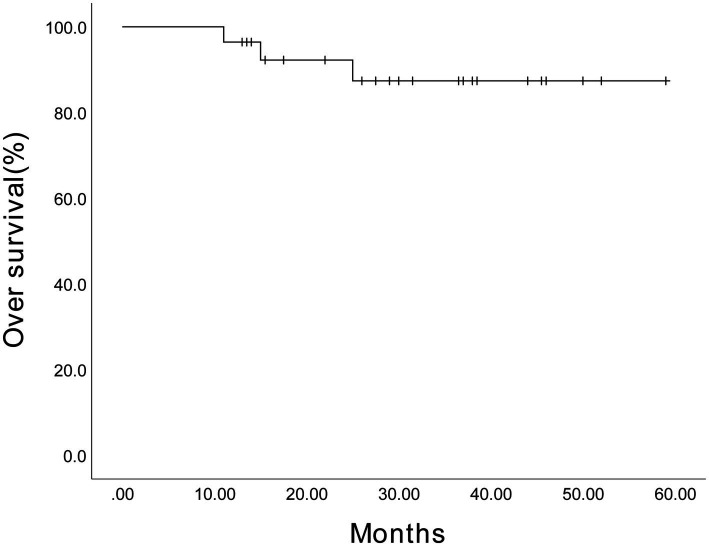
The Kaplan–Meier survival curve showing the estimated 3-year overall survival for 28 patients receiving maintenance therapy based on Ixazomib.

The estimated 3-year PFS for 64 patients was 71.2% (95% confidence interval (CI), 57.87%–84.53%) (Figure 2), and the estimated 3-year OS was 87.8% (95% CI, 79.18%–96.42%) (Figure 3). The estimated 3-year PFS for the 28 patients receiving maintenance therapy based on Ixazomib was 70.8% (95% CI, 52.18%–89.42%) (Figure 4), and the estimated 3-year OS was 87.4% (95% CI, 73.88%–100.92%) (Figure 5). The median PFS and median OS of these 28 patients were lower than those of the overall 64 patients; however, the difference was not significant (*p* > 0.05).

### 3.6 MRD status and PFS

**Figure 6:**
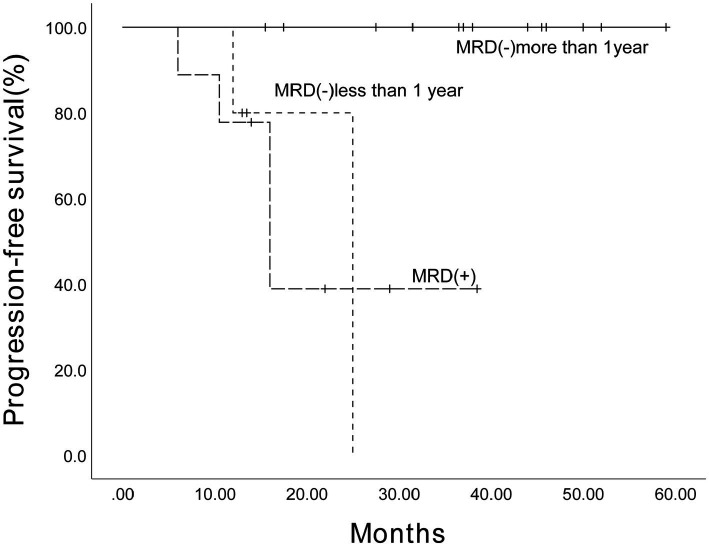
The estimated 3-year progression-free survival of 28 patients with MRD negativity (−) or positivity (+). The Kaplan–Meier survival curve was plotted. Among the 28 patients who received maintenance therapy based on Ixazomib, the estimated 3-year progression-free survival for patients who’s MRD remained persistently positive was 38.9% (95% CI, 4.99%–72.81%). For patients whose MRD negativity lasted less than 1 year, the estimated 3-year progression-free survival was 80% (95% CI, 44.92%–115.08%). In contrast, the estimated 3-year progression-free survival for patients who maintained MRD negativity for 1 year or longer was 100%.

Before ASCT, a total of 27 patients were tested for MRD, of which 10 tested positive and 17 tested negative. After ASCT, MRD testing was performed on 28 patients, with 13 testing positive. Among them, 5 achieved MRD negativity following maintenance treatment with Ixazomib. Fifteen patients were MRD-negative, with 1 patient converting to positive during follow-up. There were 9 patients whose MRD remained persistently positive, with an estimated 3-year PFS of 38.9% (95% CI, 4.99%–72.81%) (Figure 6), of which 5 relapsed (1 case of marrow relapse, 2 cases of extramedullary relapse, and 2 cases of biochemical relapse). Five patients maintained MRD negativity for less than 1 year, with an estimated 3-year PFS of 80% (95% CI, 44.92%–115.08%) (Figure 6). Among them, 1 patient experienced a biochemical relapse, and another patient died due to hepatitis-related liver failure. Eighteen patients maintained MRD negativity for 1 year or longer, with an estimated 3-year PFS of 100% (Figure 6). Within this group, 11 patients maintained MRD negativity for over 2 years, with the longest duration reaching 4.5 years, and no relapses were reported. The PFS of patients with persistent MRD positivity was significantly shorter than that of patients who achieved MRD negativity for 1 year or longer (*p* < 0.001), as illustrated in Figure 6.

### 3.7 Effect of different clinical features on PFS

**Figure 7:**
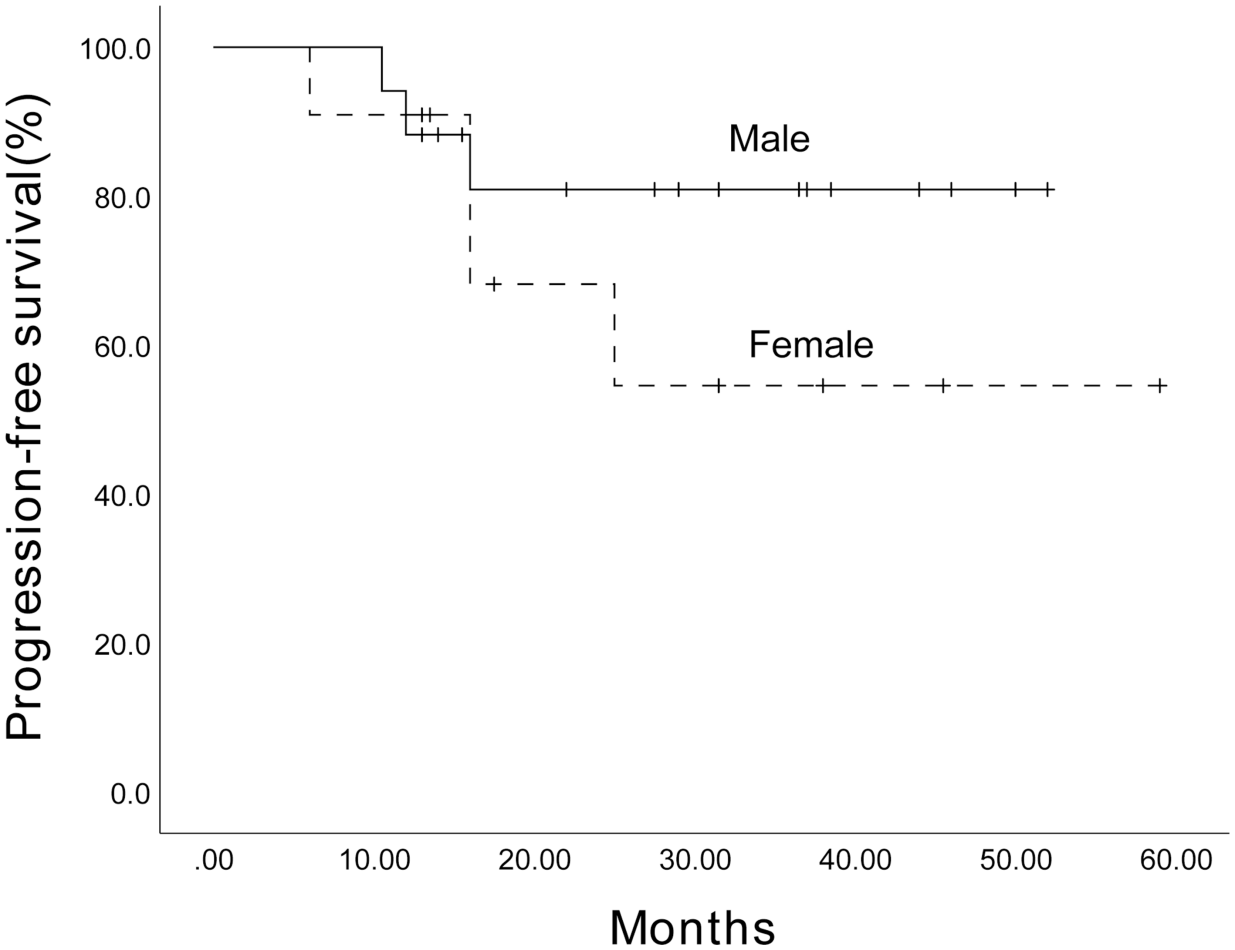
The estimated 3-year progression-free survival of the 17 male patients and 11 female patients among 28 patients receiving Ixazomib-based maintenance therapy. The Kaplan–Meier survival curve was plotted. The estimated 3-year progression-free survival was 80.9% (95% CI, 61.3%–100.5%) for males and 54.5% (95% CI, 20.6%–88.4%) for females.

**Figure 8:**
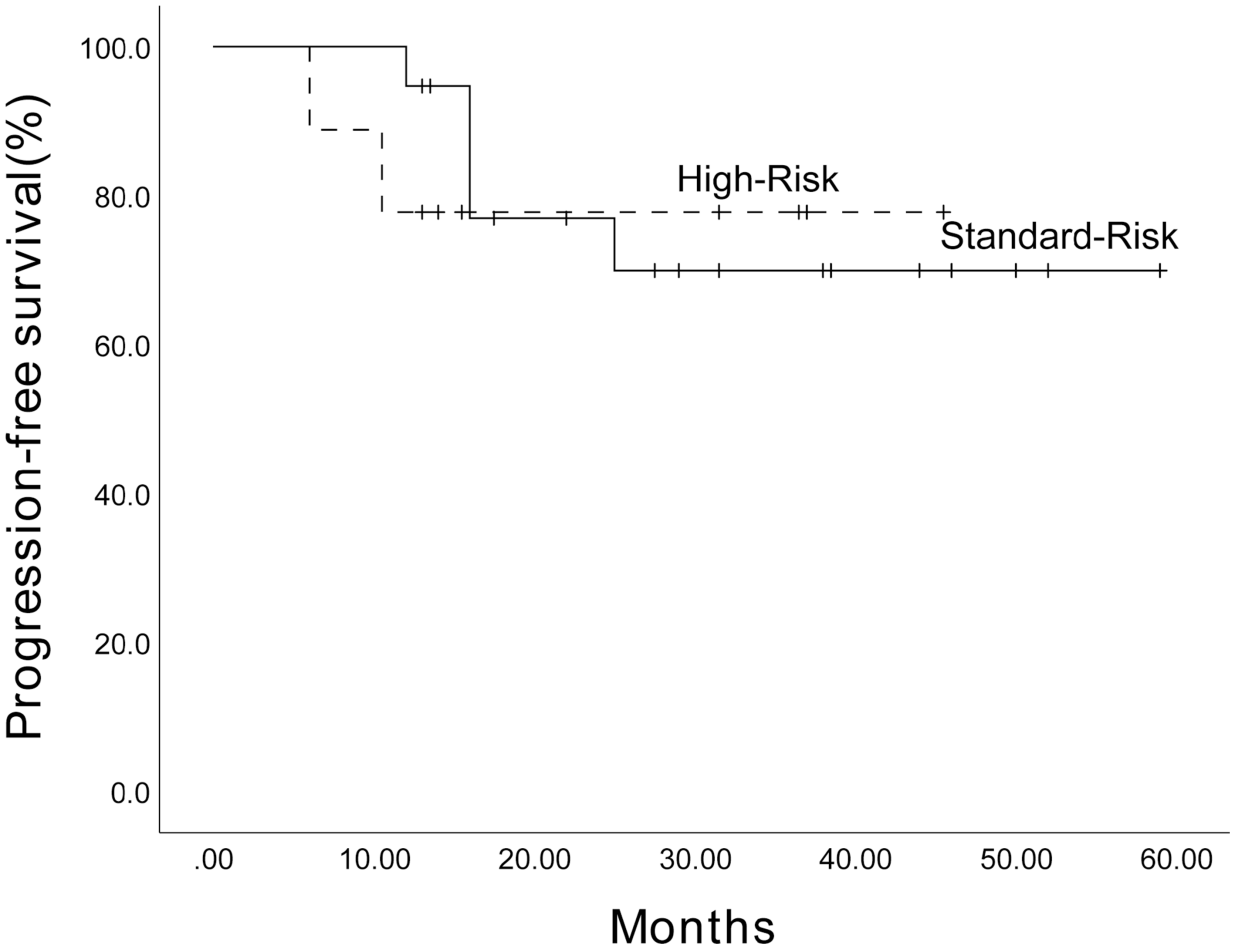
The estimated 3-year progression-free survival for patients with SR and HRCA among the 28 patients receiving Ixazomib-based maintenance therapy. The Kaplan–Meier survival curve was plotted. The estimated 3-year progression-free survival was 62.3% (95% CI, 55.05%–88.75%) for SR patients and 79.4% (95% CI, 65.76%–90.3%) for HRCA patients.

Given the limited sample size, we performed an exploratory analysis of PFS in 28 patients with MM. As presented in Figure 7, the estimated 3-year PFS rate for 17 male patients was 80.9% (95% CI, 61.3%–100.5%), while for 11 female patients, it was 54.5% (95% CI, 20.6%–88.4%), with no statistically significant difference (*p* = 0.293). Furthermore, the estimated 3-year PFS rate for 11 SR patients was 62.3% (95% CI, 55.05%–88.75%), while for 17 HRCA patients, it was 79.4% (95% CI, 65.76%–90.3%) (Figure 8). No statistically significant difference was found (*p* = 0.825). However, these comparisons are notably underpowered, and the observed numerical differences should be interpreted with caution and are considered descriptive rather than conclusive.

## 4 Discussion

The post-ASCT maintenance therapy landscape for MM is in a period of rapid and transformative change. This single-center, retrospective study evaluated the real-world efficacy and safety of Ixazomib-based maintenance therapy, conducted within the specific context of contemporary Chinese clinical practice. Our analysis of 28 patients demonstrated that this approach was feasible, with a favorable safety profile (≥3 grade adverse event incidence of 3.6%) and encouraging survival outcomes, yielding an estimated 3-year PFS of 70.8% and OS of 87.4%. These survival rates align with outcomes reported in contemporary real-world cohorts undergoing ASCT (28). Notably, sustained MRD negativity for over 1 year was associated with a 3-year PFS of 100%, underscoring its critical prognostic importance.

The established cornerstone of post-ASCT maintenance has long been lenalidomide monotherapy, supported by extensive evidence demonstrating significant improvements in PFS and OS (13, 14, 29, 30). Recently, this standard has been elevated by the integration of monoclonal antibodies. The phase III PERSEUS trial established Dara-R as a new, more potent standard of care for transplant-eligible patients, showing superior PFS and deeper MRD responses compared to lenalidomide alone (15). Similarly, the AURIGA study confirmed that Dara-R effectively converted MRD-positive patients to negativity post-ASCT (31). However, the immediate global adoption of such regimens is constrained by regional access and reimbursement policies. In China, daratumumab is not yet approved or reimbursed for post-ASCT maintenance, creating a significant gap between ideal care and practical, sustainable therapy for most patients. Furthermore, survival outcomes for patients with HRCA remain unsatisfactory compared to patients with SR, with a substantial proportion experiencing early relapse despite modern therapy (32). Therefore, the development of effective, accessible, and sustainable maintenance strategies for HRCA patients is an urgent clinical priority (16). In this context, our findings regarding Ixazomib are highly pertinent. The oral administration and favorable neurotoxicity profile of Ixazomib offer distinct practical advantages over injectable proteasome inhibitors like bortezomib for long-term use (20, 21). This advantage is supported by studies demonstrating the efficacy and tolerability of fully oral Ixazomib-containing regimens across different treatment settings (33–35). While analyses of the TOURMALINE-MM3 trial confirmed a significant yet modest PFS benefit for single-agent Ixazomib versus placebo (19), the head-to-head randomized comparison (MMRC-066) suggested its efficacy might be suboptimal compared to lenalidomide maintenance (18). Consequently, Ixazomib’s most relevant clinical role lies as a viable alternative for patients intolerant to lenalidomide, or as a rational component of combination strategies, particularly for high-risk disease where proteasome inhibitor-based maintenance is recommended (16, 17).

Our observations align with a growing body of contemporary evidence. Real-world studies from Germany and the Nordic region have reported 2-year PFS rates of 64% and 66%, respectively, with Ixazomib-based maintenance, supporting its activity and feasibility in routine practice (23, 24). The UK Myeloma XII (ACCoRD) trial further demonstrated that Ixazomib consolidation and maintenance significantly extended PFS compared to observation in the salvage ASCT setting (36). Importantly, the favorable tolerability profile noted in our cohort is corroborated by the phase II MMRC-066 study, which reported a lower rate of dose reductions with Ixazomib compared to lenalidomide maintenance (9% vs. 24%) (18). Collectively, these international data frame our results as part of an ongoing reassessment of Ixazomib’s utility within modern, risk-adapted maintenance approaches.

A central and robust finding of our study is the dominant prognostic value of sustained MRD negativity. Patients who maintained MRD-negative status for one year or longer had an exceptional 3-year PFS of 100%, in stark contrast to those with persistent MRD positivity (38.9%). This aligns perfectly with large-scale prospective analyses. A pooled analysis of the TOURMALINE-MM3 and -MM4 trials definitively showed that achieving and sustaining MRD negativity on Ixazomib maintenance was a key determinant of superior long-term outcomes (19). Our data reinforce this principle in a real-world setting and suggest that Ixazomib-based regimens can contribute to attaining this critical treatment goal, which may help mitigate the negative prognostic impact of adverse cytogenetics (37).

This study has several important limitations that must be acknowledged, consistent with its hypothesis-generating nature. First, the statistical power is constrained by the single-center, retrospective design and the limited sample size. This precludes robust subgroup analyses and definitive conclusions, particularly regarding comparative efficacy in high-risk patients, which would require a much larger, prospectively defined cohort. Second, the non-randomized assignment to treatment regimens introduces potential selection bias, although we have detailed the clinical rationale for these choices. Third, the median follow-up of 28.5 months may be insufficient to fully assess long-term outcomes and late-onset toxicities. Fourth, MRD assessment was performed using first-generation flow cytometry with a sensitivity of 10^−5^, which is less sensitive than next-generation flow or sequencing. This may have led to an overestimation of the MRD-negative population, and the prognostic strength of MRD negativity we observed should be interpreted within this methodological context.

In conclusion, within the practical constraints of the current Chinese healthcare environment, Ixazomib-based maintenance therapy demonstrates good efficacy and a favorable safety profile for MM patients after ASCT. It represents a viable, accessible, and reimbursed option for patients ineligible for or intolerant to lenalidomide, and serves as a rational component of combination strategies for high-risk disease. The profound prognostic impact of sustained MRD negativity remains a dominant theme, underscoring its value as a treatment goal and dynamic monitoring tool. Future prospective, multicenter studies with larger sample sizes and more sensitive MRD techniques are warranted to definitively establish the role of Ixazomib within contemporary, risk-adapted maintenance paradigms.

## Data Availability

The raw data supporting the conclusions of this article will be made available by the authors, without undue reservation.
